# Pneumococcal Transmission and Disease *In Silico*: A Microsimulation Model of the Indirect Effects of Vaccination

**DOI:** 10.1371/journal.pone.0056079

**Published:** 2013-02-06

**Authors:** Markku Nurhonen, Allen C. Cheng, Kari Auranen

**Affiliations:** 1 Department of Vaccination and Immune Protection, National Institute for Health and Welfare, Helsinki, Finland; 2 Department of Epidemiology and Preventive Medicine, Monash University and Infectious Diseases Unit, Alfred Hospital, Melbourne, Victoria, Australia; Health Protection Agency, United Kingdom

## Abstract

**Background:**

The degree and time frame of indirect effects of vaccination (serotype replacement and herd immunity) are key determinants in assessing the net effectiveness of vaccination with pneumococcal conjugate vaccines (PCV) in control of pneumococcal disease. Using modelling, we aimed to quantify these effects and their dependence on coverage of vaccination and the vaccine's efficacy against susceptibility to pneumococcal carriage.

**Methods and Findings:**

We constructed an individual-based simulation model that explores the effects of large-scale PCV programmes and applied it in a developed country setting (Finland). A population structure with transmission of carriage taking place within relevant mixing groups (families, day care groups, schools and neighbourhoods) was considered in order to properly assess the dependency of herd immunity on coverage of vaccination and vaccine efficacy against carriage. Issues regarding potential serotype replacement were addressed by employing a novel competition structure between multiple pneumococcal serotypes. Model parameters were calibrated from pre-vaccination data about the age-specific carriage prevalence and serotype distribution. The model predicts that elimination of vaccine-type carriage and disease among those vaccinated and, due to a substantial herd effect, also among the general population takes place within 5–10 years since the onset of a PCV programme with high (90%) coverage of vaccination and moderate (50%) vaccine efficacy against acquisition of carriage. A near-complete replacement of vaccine-type carriage by non-vaccine-type carriage occurs within the same time frame.

**Conclusions:**

The changed patterns in pneumococcal carriage after PCV vaccination predicted by the model are unequivocal. The overall effect on disease incidence depends crucially on the magnitude of age- and serotype-specific case-to-carrier ratios of the remaining serotypes relative to those of the vaccine types. Thus the availability of reliable data on the incidence of both pneumococcal carriage and disease is essential in assessing the net effectiveness of PCV vaccination in a given epidemiological setting.

## Introduction

*Streptococcus pneumoniae* (pneumococcus) is a commensal that colonises the human nasopharynx. Colonisation occurs as episodes of carriage, starting with the acquisition of a pneumococcus clone, followed by a stable period of carriage until the clearance of the bacterium. Only rarely does carriage lead to infections of the mucosa (e.g. otitis media and pneumonia) or systemic infections (invasive pneumococcal disease). The polysaccharide capsule is the main virulence factor [1], and pneumococci are classified into more than 90 serotypes based on the antigenic properties of the capsule [2]. The frequency of different serotypes in carriage and their propensity to cause invasive disease per episode of carriage vary significantly [3], [4].

The first pneumococcal conjugate vaccine (PCV7) containing antigens against 7 pneumococcal serotypes was licensed for use in routine childhood immunisation in the USA in 2000. More recently, 10-valent (PCV10) and 13-valent (PCV13) vaccines have become available and adopted in immunisation programmes in large parts of the developed world and in some developing countries [5]. The direct protective effect of PCV against invasive pneumococcal disease (IPD), pneumonia, and otitis media caused by the vaccine serotypes has been demonstrated in a number of clinical trials. While vaccine efficacy against vaccine-type IPD is very high (90%), it is considerably lower against pneumonia and otitis media [6]. Vaccine-induced protection is largely confined to vaccine serotypes (VT).

In addition to protecting against VT disease, a large-scale use of PCV reduces the risk of VT carriage in vaccinated individuals [7], [8], thus reducing VT circulation in the population at large. This induces two major types of indirect effects of vaccination. The unvaccinated population gains protection against VT carriage and disease due to *herd immunity*. The other indirect effect is *serotype replacement* in which the reduced VT circulation opens a niche for non-vaccine serotypes (NVT), increasing the incidence of NVT carriage and potentially also NVT disease. The two indirect effects work in opposite directions in determining the net effectiveness of PCV vaccination [9].

There are important public health considerations regarding herd immunity and serotype replacement. An obvious question concerns the degree of indirect protection induced by a planned vaccination programme. The economic evaluation of PCV vaccination depends heavily on the inclusion of indirect effects in the analysis [10]. More generally, to understand the conditions for VT elimination, it is necessary to explore how indirect protection depends on the coverage of vaccination and the vaccine efficacy against VT carriage. Near-elimination of VT carriage and disease has occurred in populations under broad-scale vaccination with PCV [11], [12]. At the same time, the replacement of VT disease by NVT disease has partially offset the benefits of vaccination, with varying reports on its magnitude [13]. However, transmission dynamic models have not been used extensively to address conditions for VT elimination and the extent of serotype replacement in carriage and disease.

Serotype replacement in pneumococcal carriage appears to be complete and take place readily after the onset of a vaccination programme [14], [15]. The obvious underlying mechanism of replacement is competition among pneumococcal strains and serotypes [1], [16], [17]. In mathematical models of pneumococcal transmission, competition has invariably been quantified as reduced acquisition of pneumococcal carriage in an individual already carrying another serotype. The problem with a large number of serotypes has been tackled through pooling of serotypes into two or three aggregate types by their inclusion in the vaccine formulation (VT/NVT) [18], [19], antibiotic susceptibility [20], or capsular switch status [21]. The only studies accounting for a larger number of types in a dynamic model appears to be Van Effelterre et al. [22], which addresses the impact of PCV on antibiotic resistance and IPD in children <2 years of age, and Cobey et al. [23], which investigates mechanisms that maintain diversity in pneumococcal serotypes in unvaccinated and vaccinated populations. In addition, the net effectiveness of pneumococcal vaccination has been considered using non-dynamic cohort models [9], [24]. However, cohort models cannot adequately address all implications of indirect effects of vaccination.

In this article, we introduce a comprehensive transmission dynamic model of pneumococcal carriage and disease. The model is based on a microsimulation of the demography and carriage in social mixing groups relevant for pneumococcal transmission in a developed country and incorporates dynamic modelling of a large number of competing serotypes. Progression of carriage to invasive pneumococcal disease is considered in terms of serotype-specific attack rates (case-to-carrier ratios). The main aim of this study was to examine the net effectiveness of PCV vaccination, i.e., the extent of herd immunity against VT carriage and IPD, and that of replacement IPD, over time. We compare the effectiveness of vaccination under different serotype compositions in the vaccine.

## Methods

### Stratification of serotypes and model parameterisation

**Figure 1:**
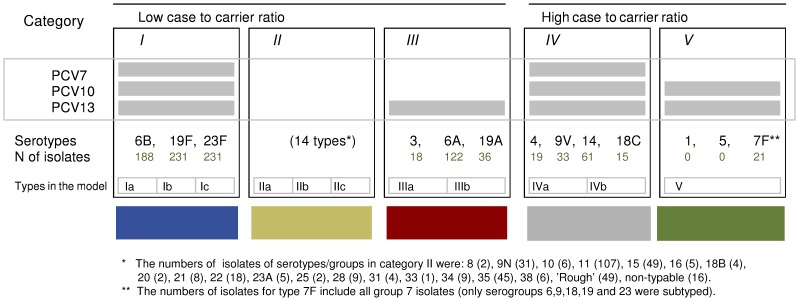
Stratification of pneumococcal serotypes. Serotypes are stratified into 5 categories, based on their inclusion in different PCV formulations (PCV7, PCV10 and PCV13) and their case-to-carrier ratios. The number (*N*) of isolates refers to the distribution of serotypes in children <2 years of age [25]. In the model, each category is subdivided into a given number of individual model types.

Figure 1 presents a stratification of pneumococcal serotypes into five epidemiologically distinct categories, based on their inclusion in different PCV formulations and their propensity to cause invasive disease. The stratification serves as a basis to parameterise competition among serotypes, enabling predictions of the long-term effects of vaccination in terms of individual serotypes. The categories are labelled from I to V in a decreasing order of prevalence in carriage.

The currently available PCV formulations are PCV7 (serotypes 4, 6B, 9V, 14, 18C, 19F and 23F), PCV10 (additional serotypes 1, 5, 7F), and PCV13 (additional serotypes 3, 6A, 19A). Vaccine types (VT)/non-vaccine types (NVT) refer to serotypes included/not included in the vaccine. Note that a vaccine with a higher valency always includes all serotypes in a vaccine with a lower valency.

The classification of the PCV7 types into two categories (I and IV) with low and high case-to- carrier ratios (probability of disease per carriage episode), respectively, was initially based on Brueggemann et al. [4], who considered the ratio of IPD incidence to the point prevalence of carriage by serotype. This division of the PCV7 types as well as labelling of the rest of categories as corresponding to low or high case-to-carrier ratios proves to agree with the data in the current analysis as well (see below).

For parsimonious modelling, each of the five serotype categories was divided into one or more *model types*, which were treated individually but assumed to share identical rates of acquisition and clearance. The model types do not necessarily correspond to individual serotypes. The number of model types within each category was chosen so that a model type in a category with a higher rank has a higher prevalence than any model type in a lower-ranked category. The mutual ranking of model types within any given category is arbitrary. The ranking corresponds to model types with higher ranks (i.e., higher prevalence) having larger competitive strengths than model types with lower ranks (see below). The total number of model types is 11.

### Empirical data

#### Age-specific prevalence of pneumococcal carriage

**Figure 2:**
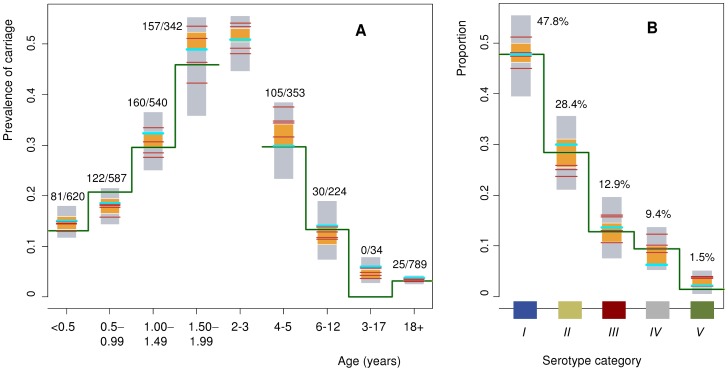
Age-specific prevalence of carriage and the serotype distribution. (A) The prevalence of pneumococcal carriage in 9 age windows (<0.5, 0.5–0.99, 1.00–1.49, 1.50–1.99, 2–3, 4–5, 6–12, 13–17, 18+ years of age). The blue and red horizontal lines correspond to the model simulation using the 5 most optimal parameter combinations having the 5 highest (median) likelihood values, with the blue lines indicating the most optimal combination (Table 1). The ranges of model predictions are shown for each age window in grey colour. These correspond to the 50 most optimal parameter combinations. The point-wise intervals containing 75% of these 50 prevalence values are shown in yellow. The observed proportions (number of positive samples/number of samples) are indicated by the green line segments (there were no observations in age window 2–3 years). These data originate from 3 studies as described in the text. (B) The serotype distribution for the five serotype categories. The simulation results and the observed data are presented as in panel A. The observed data originate from a study of pneumococcal carriage in children <2 years of age (Table 1; [25]).

Figure 2A presents data about the prevalence of pneumococcal carriage by age. The data were derived from various sources as described in the following. The prevalence of pneumococcal carriage in 329 unvaccinated Finnish children <2 years of age was recorded in the FinOM Cohort Study [25]. The data were originally stratified according to the health status of the child at the time of sampling (healthy vs. sick visits). To describe the natural history of carriage in young children, only one sample of either type was taken into account for each child at each of the eight four-week age windows (at 2,5,6,9,12,15,18, and 24 months of age). The prevalence of pneumococcal carriage increases from 13% at 2 months of age to 46% at 24 months of age.

Pneumococcal carriage in unvaccinated children and adults was recorded in family members of day care attendees during a 9 month follow-up in Finland [26]. The mean prevalence of carriage in 6–12 year old siblings of day care attendees was 13% (30/224), in 13–17 year old siblings 0% (0/34), and in adult family members (median age 36 years) 3% (25/789). The prevalence of pneumococcal carriage was 30% (105/353) in 4–5 year old children that had participated in a vaccine trial 3 years earlier [27].

#### Serotype distribution in carriage

Figure 1 lists the numbers of isolates of individual serotypes in children less than 2 years of age in the FinOM Cohort data [25]. Based on these data, Figure 2B presents the serotype distribution aggregated according to the five serotype categories.

### The demographic model

The demographic model has been described in detail elsewhere [28] (see also Table S1.1 in File S1). Briefly, it is an individual-based contact network (microsimulation) model that mimics the population turnover of the population in a developed country (Finland). The mixing pattern involves contacts within families, two different types of day care groups (1–2 years of age, 3–6 years of age; 44% of children attending), school classes and the general population. In addition, a sixth level of mixing, neighbourhoods, was now implemented by partitioning the total simulated population of 100,000 individuals into 20 neighbourhoods of size 5,000. The microsimulation of the demography readily produces a realistic pattern of mixing groups in the Finnish population.

### The epidemiological model of pneumococcal transmission

**Figure 3:**
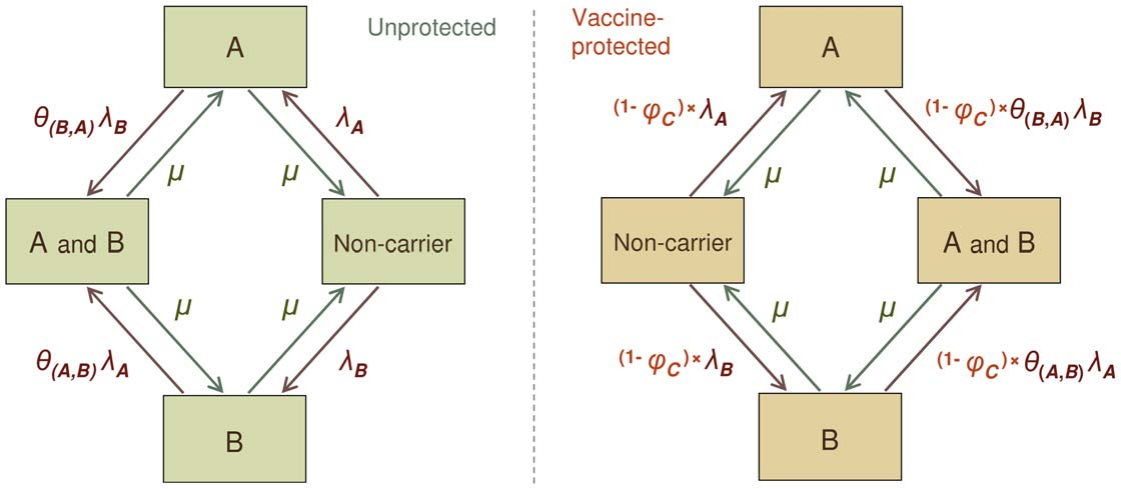
Model for transmission of pneumococcal carriage. Each individual in the population belongs to one of 67 possible states: susceptible to carriage, carrier of one of the 11 model types, or simultaneous carrier of any two of the 11 model types. For simplicity, the figure presents the model for two of the 11 model types, denoted as A and B. The flow of individuals is governed by rates of clearance and acquisition, as marked at each arrow. The rates of acquisition depend on the number of carriers in the mixing group which the individual belongs to and, for already carrying individuals, also on the ranking order of the categories corresponding to the incoming and the carried type (see text). The rates of acquisition for vaccine-protected individuals are reduced by a factor (1-*φ_c_*). Vaccinated individuals lose protection against carriage with a waning rate (see text) and move to an unprotected compartment. The rate of clearance *μ* is assumed same, regardless of the vaccination status, the type(s) and the number of types carried.

At any time, an individual is either a non-carrier (susceptible), carrier of one of the 11 model types, or a double carrier of two different model types. A schematic presentation of these epidemiological states is presented by Figure 3. Transitions between these states are governed by rates of acquisition and clearance as described in the following.

#### Acquisition of carriage

For individual *i*, the number of individuals and the number of carriers of type *j* in his/her *k*th social mixing group at time *t* are denoted by *N_ik_(t)* and 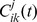, respectively, *k* = 1,…,6; *j* = 1,…,11. The per capita rate at which a non-carrying (susceptible) individual of age a acquires carriage of type *j* at time *t* is defined as
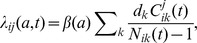
where *β(a)* is the age-dependent baseline rate of acquisition and *d_k_*, *k* = 1, 2,…,6, are the six mixing group-specific relative contact rates of acquisition (Table 1). The rate of acquisition for a particular type thus depends on the numbers of carriers of the type in the mixing groups of the individual. Double carriers are assumed to be equally infectious for both carried types. To avoid extinction, if the total number of carriers of any model type *j* was below 50, say 50-*x*, a supplementary force of infection corresponding to *x* carriers in the population was added to λ*ij*(*a*,*t*).

**Table 1:** Parameter values.

	Parameter type	Parameter	Value (grid range)	Source
(A)	Relative rates of acquisition	Family, *d*_1_	1.2 (0.6, 1.5)	Calibration parameter
		Day care 1, *d*_2_	0.6 (0.2, 1.4)	Calibration parameter
		Day care 2, *d*_3_	1.2 (0.8, 2.2)	Calibration parameter
		School, *d*_4_	0.80 (0.25, 1.20)	Calibration parameter
		Neighbourhood, *d*_5_	0.133 (0.083, 0.200)	Calibration parameter
		Population, *d*_6_	*d*_5_/2	Ratio *d*_6_/*d*_5_ fixed to 0.5
	Baseline rates of acq. (per day)	Age group [0,1), *β*_1_	0.045	For definiteness
		Age group [1,4), *β*_2_	0.070	Fixed value
		Age group 4+ y., *β*_3_	0.020	Fixed value
	Competition parameters	*θ*_1_	0.800	Fixed value
		*θ*_2_	0.744	Fixed value
(B)	Rates of clearance	Children under 5 y.	0.67 per month	[29], [30]
		Individuals 5+ y.	1.00 per month	[29], [30]

(A) Parameter values that were assessed in the context of the simulation model. The age-specific baseline rate of acquisition of pneumococcal carriage, *β*(*a*), was considered in 3 age groups as a stepwise function with values *β*_1_, *β*_2_ and *β*_3_. There are 6 mixing group-specific parameters (*d*_1_–*d*_6_) in the model, corresponding to family, 2 different types of day care groups (children 0.5–2 years of age, and children 3–6 years of age), school (children 7–17 years of age), neighbourhood, and the general population. Five of these (*d*_1_–*d*_5_) were calibrated. A range of the design grid (in parentheses) is given that covers values corresponding to the 9,000 simulation runs in the calibration process. The values of the two competition parameters were searched before the final calibration phase so that their ratio (*θ*_2_/*θ*_1_ = 0.93) reproduces the serotype distribution. For more details, see File S2. (B) Parameter values that were given literature values.

Between-type competition in colonisation was included in the model by means of a model type dependent acquisition rate for double carriage in an already carrying individual. This rate, given by *θ(h,j)λ_ij_*(*a,t*), where *θ(h,j)*<1 is the parameter accounting for the reduced acquisition rate for double carriage, depends on the ranks of the currently colonising type *h* and of the incoming type *j* in the hierarchy of the model types. Specifically, parameter *θ(h,j)* is assigned one of two possible values, *θ*_1_ or *θ*_2_, satisfying the constraints 0<*θ*_2_<*θ*_1_<1. For an incoming model type with a higher (lower) rank than the currently carried model type, *θ(h,j)* = *θ*_1_ (*θ(h,j)* = *θ*_2_). The quantity 1- *θ*_2_/*θ*_1_ is related to the skewness of the serotype distribution. In particular, *θ*_2_ = *θ*_1_ would correspond to symmetric competition, i.e., a uniform distribution across the model types.

#### Clearance of carriage

We assume a constant rate of clearance with an exponential distribution for the duration of carriage, with the rate of 0.67 per month in children <5 years of age. The rate is assumed to be 1.5 times higher in individuals of age 5+ [29], [30].

#### Serotype distribution in disease and the case-to-carrier ratios

**Figure 4:**
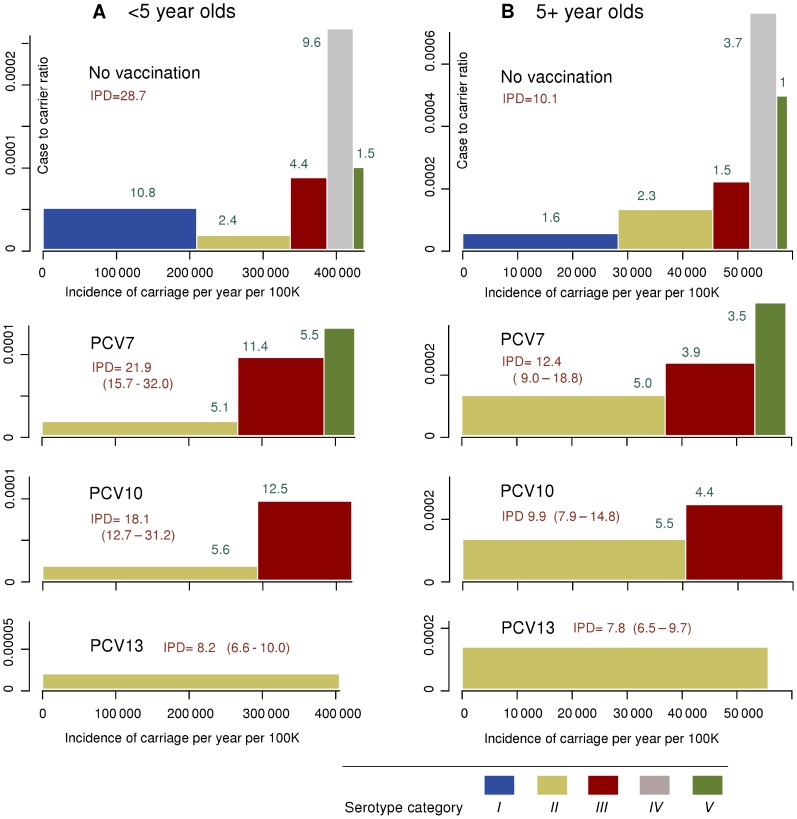
Incidence of carriage and case-to-carrier ratios by serotype category in two age classes in Finland. The stationary incidence of pneumococcal carriage (per year per 100,000 persons) is indicated on the horizontal axis by category, shown cumulatively. The serotype category-specific case-to-carrier ratios are shown on the vertical axis; note the different scales in the <5 and 5+ panels. The area of each rectangle represents the annual IPD incidence (per 100,000) in the respective serotype category. The upmost horizontal pair of panels shows the annual IPD incidence (per 100,000) before vaccination. The category-specific incidences of carriage (horizontal axis) correspond to the average pre-vaccination incidences. The other panels correspond to the projected IPD incidences after the onset of vaccination programme with PCV7, PCV10 and PCV13. The projected overall and serotype category-specific IPD incidences (indicated in green font) are obtained as averages from simulation runs based on the 50 most optimal parameter combinations. The post-vaccination panels show the average projected IPD incidence (area), the average case-to-carrier ratios (vertical axis) and the corresponding incidence of carriage (horizontal axis). The pairs of values in parentheses are the corresponding ranges of projected IPD incidences based on the 50 simulation runs.

The incidence of invasive pneumococcal disease (IPD) in Finland was collected from the years 1995–2006, stratified by age class and serotype category. For each age class and serotype category, the case-to-carrier ratio, i.e., the probability of carriage episode to progress to IPD was calculated as the ratio of the respective average annual IPD incidence to the stationary incidence of carriage episodes. The incidence of carriage was extracted from the simulation output. The case-to-carrier ratios are shown on the vertical axis of Figure 4 for age classes <5 and 5+. Note that the case-to-carrier ratios in categories I–III are “low” and those in categories IV–V “high”, in agreement with the terminology adopted in Figure 1. The actual annual mean incidence of IPD is the area of the respective rectangle.

#### Sensitivity of detection of carriage

The empirical data available in this study only record one serotype per carrier. To link model predictions to data, the sensitivity of detecting carriage in both single and double carriers was taken to be 100%. In a single carrier, the carried serotype was always detected. In a double carrier, either of the two types was detected as the carried one, both having a 50% probability of detection.

#### Structure of transmission

The implied structure of transmission can be characterised by the number of secondary infections produced by a typical infective in the structured population during a single episode of carriage. This is obtained as the leading eigenvalue of the basic reproduction matrix *K* (see e.g. [31]). The entry (*i*,*j*) of matrix *K* is the number of infectious contacts from one carrier in stratum *j* to individuals in stratum *i*. The stratification is based on 29 age groups, presence of siblings under 7 years of age (yes/no for children <7 years of age), parental status (with/without children <7 years of age), day care attendance (yes/no), resulting in a total of 55 strata. The elements of *K* are thus given by the expression
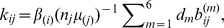
where 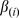 is the age-specific baseline rate of acquisition in stratum *i*, *n_j_* is the number of individuals in stratum *j*, and *μ*_(*j*)_ is the age-specific rate of clearance in stratum *j*. Furthermore, *B* ^(*m*)^ is the symmetric mixing matrix specific to the mixing group. The element 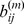 of *B* ^(*m*)^ is the sum of contact contributions from all individuals in stratum *j* that have contacts to individuals in stratum *i* in mixing group of type *m* (e.g. family). For each such individual in stratum *j*, the contact contribution is defined as 1/*M* where *M* is the number of all other individuals sharing the same mixing group, cf. [28] [32]. Matrix *B* ^(*m*)^ is calculated from one simulation run of the demographic model for a population of size 100,000, independently of the infection model. The basic reproduction matrix *K*, corresponding to a single model type in the absence of other types, is the sum of the 6 mixing group-specific reproduction matrices obtained in this manner.

The matrix of average number of transmission events per time unit in the population by source stratum (columns) and recipient stratum (rows) is then (*I*-*P*)*KNUP*, where *P* is the diagonal matrix of carriage prevalences in the 55 strata, *I* is the identity matrix, *N* is the diagonal matrix of numbers of individuals in each stratum and *U* is the diagonal matrix of age-specific rates of clearance *μ*_(*j*)_, *j* = 1, 2, …, 55.

### Vaccination

The impact of vaccination depends on the coverage of vaccination and on vaccine efficacies for susceptibility to carriage and progression of carriage to disease. In the model, a proportion *p_c_* of children completes a full regime of PCV vaccination at the age of one year. Before that the child is assumed to be fully susceptible to carriage and disease. After one year of age, a vaccinated child is protected against carriage and disease according to vaccine efficacies as specified below. We explore the consequences of different levels of the coverage *p_c_* of vaccination on the elimination thresholds for pneumococcal carriage.

There are two vaccine efficacy parameters in the model. Vaccine efficacy against pneumococcal acquisition (*φ_c_*) is the relative percentage reduction in the rate of acquisition of carriage, due to the individual being vaccinated. The same efficacy against acquisition is assumed for all vaccine types. We consider the effect of different levels of efficacy on the elimination thresholds for pneumococcal carriage. Vaccine efficacy against progression to disease (*φ_D_*) is the relative percentage reduction in the probability of IPD per episode of carriage, due to the individual being vaccinated. In each scenario, the same value (*φ_D_* = 90%) was used for all vaccine types.

In addition, the efficacy of vaccine may wane over time. In lack of clear evidence about this, we used values 10% and 25% per year (the rate of loss of vaccine-induced protection), corresponding to an average of 10 and 4 years protection against carriage, respectively. This waning is considered only with regard to efficacy against carriage. Of note, assumptions about waning immunity against disease are irrelevant in the current analysis, as the model predictions about disease only pertain to the new steady state when vaccine-type carriage and disease are eliminated. The possibility of vaccine-induced cross-immunity against serotypes not included in the vaccine formulation is not considered.

### Calibration of the simulation model

Empirical data on the age-specific prevalence of pneumococcal carriage (Figure 2A) and the serotype distribution (Figure 2B) prior to PCV vaccinations were used to estimate parameters that determine pneumococcal transmission. For details of model calibration, see File S2. Briefly, values for 11 parameters, listed in Table 1 (part A), were assessed. In particular, the 5 mixing group-specific relative rates were optimised to maximise a likelihood function, which measures the concordance of the model output to the data. The most optimal parameter combinations were defined as those corresponding to the 50 largest median likelihood values, see Text S2.1 and Figures S2.1 and S2.2 in File S2. Model projections were realised using the optimal parameter combinations. In addition, we define a range of plausible values for a quantity of interest as those based on the set of the 50 most optimal parameter combinations (cf. Garnett et al. [33]). The simulation code was implemented using C++ and run on a cluster of 250 Intel Xeon 2.27 GHz processors.

In addition to parameters that were based on the empirical data described above, some parameters of the simulation model were based on values reported in the literature (Table 1, part B). These include the age-specific rates of clearing carriage (see above). Finally, when different scenarios of vaccination were considered, the vaccine coverage (*p_C_*) and vaccine efficacy against acquisition (*φ_c_*) were treated as *control parameters*, i.e., they were assigned ranges of alternative values in producing model predictions.

## Results

### Model calibration and validity

The simulation model was calibrated by searching a set of values for the 11 model parameters (File S2 and Table 1). Figure S2.1 in File S2 presents the approximate profile likelihood for the 5 mixing group-specific parameters. As could be expected, there were some dependencies among the supported values. In particular, there is a clear negative dependence between parameters *d*_1_ (family) and most of the other mixing group-specific rates.

The model simulations agree well with the observed pattern of the age-specific prevalence of carriage (Figure 2A), indicating that the model appropriately reproduces the incidence of colonisation. The high prevalence among children 1–4 years of age is mainly explained by their higher rate of acquisition (*β*_2_) in comparison to other age groups.

The case-to-carrier ratios were calculated as ratios of the observed IPD incidence to the incidence of carriage episodes in each of the five serotype categories and two age classes (Figure 4, upper panels). In children <5 years of age, they vary between less than 1 to 4 per 100,000 episodes of carriage, roughly in concordance with the analysis of Sleeman et al. [34]. The case-to-carrier ratios in categories I–III are “low” and those in categories IV–V “high”, in agreement with the terminology adopted in Figure 1. The actual annual mean incidence of IPD is the area of the respective rectangle. Note the importance of category I as a cause of IPD in age class <5 years of age, in contrast to the older age class.

The two competition parameters (*θ*_1_, *θ*_2_) govern the serotype distribution. The estimated value of the ratio *θ*_2_/*θ*_1_ (0.93) yields a good fit to the observed distribution of serotype categories (Figure 2B). With the choice of *θ*_1_ = 0.80, a higher-in-rank model type experiences a 20% reduction in the rate of acquisition if the individual is already colonised with a lower-in-rank type. For a lower-in-rank type, this reduction is 26% (i.e., 1−0.8×0.93) if the individual is already colonised with a higher-in-rank type. In the following, results are presented for these values (*θ*_1_ = 0.80, *θ*_2_ = 0.74), corresponding to the proportion of double carriage of approximately 23% among carriers. Results pertaining to an alternative pair of values (*θ*_1_ = 0.5, *θ*_2_ = 0.47) are presented in Figures S3.1 and S3.2 in File S3.

### Transmission structure and potential

**Figure 5:**
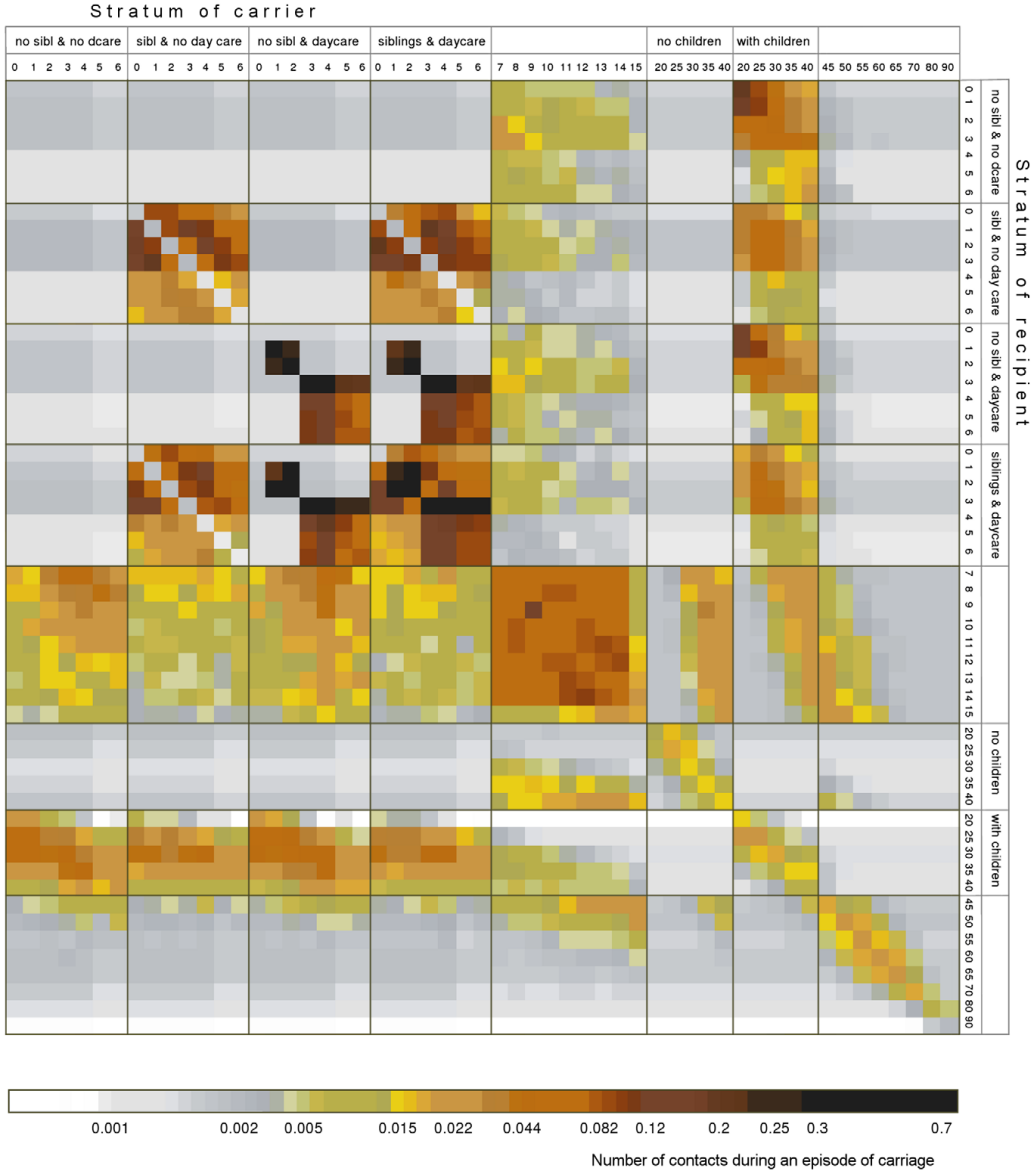
Basic reproduction matrix *K*. Each column in the plot corresponds to a single carrier of a given stratum. The stratification is based on 29 age groups, presence of siblings under 7 years of age (yes/no for children <7 years of age), parental status (with/without children <7 years of age for adults 20–45 years of age), day care attendance (yes/no). Each entry indicates the average number of infectious contacts from a single carrier in stratum *j* (column) to a one-year age cohort of individuals in stratum *i* (row). Darker colours indicate higher numbers. The numbers are calculated from the distribution of social contacts extracted from the simulation and applying the most optimal parameter combintation. The column/row numbering refers to the lower limit of the age group.

Figure 5 presents the basic reproduction matrix *K* induced by the simulation model. Based on matrix *K* and calculated for a single serotype in the absence of others and using structure of transmission stratified by age together with parenthood, sibling and day care status, the basic reproduction number is 2.0 (range of plausible values 1.9–3.7), indicating a transmission potential whose effect on the population can be expected to be significantly reduced by the application of control efforts of a reasonable magnitude. Based on the stationary age distribution of susceptibles and the basic reproduction matrix *K*, in 44% (with range of plausible values 40–61%) of transmission events, with regard to carriage, in the whole population the source is a child (<7 years of age) and 18% (range 17–31%) of transmission events occurs among <7 year old children (see Table S1.2 in File S1). This pattern highlights the importance of children in the transmission of pneumococcal carriage.

### Elimination of vaccine-type carriage and serotype replacement in carriage

**Figure 6:**
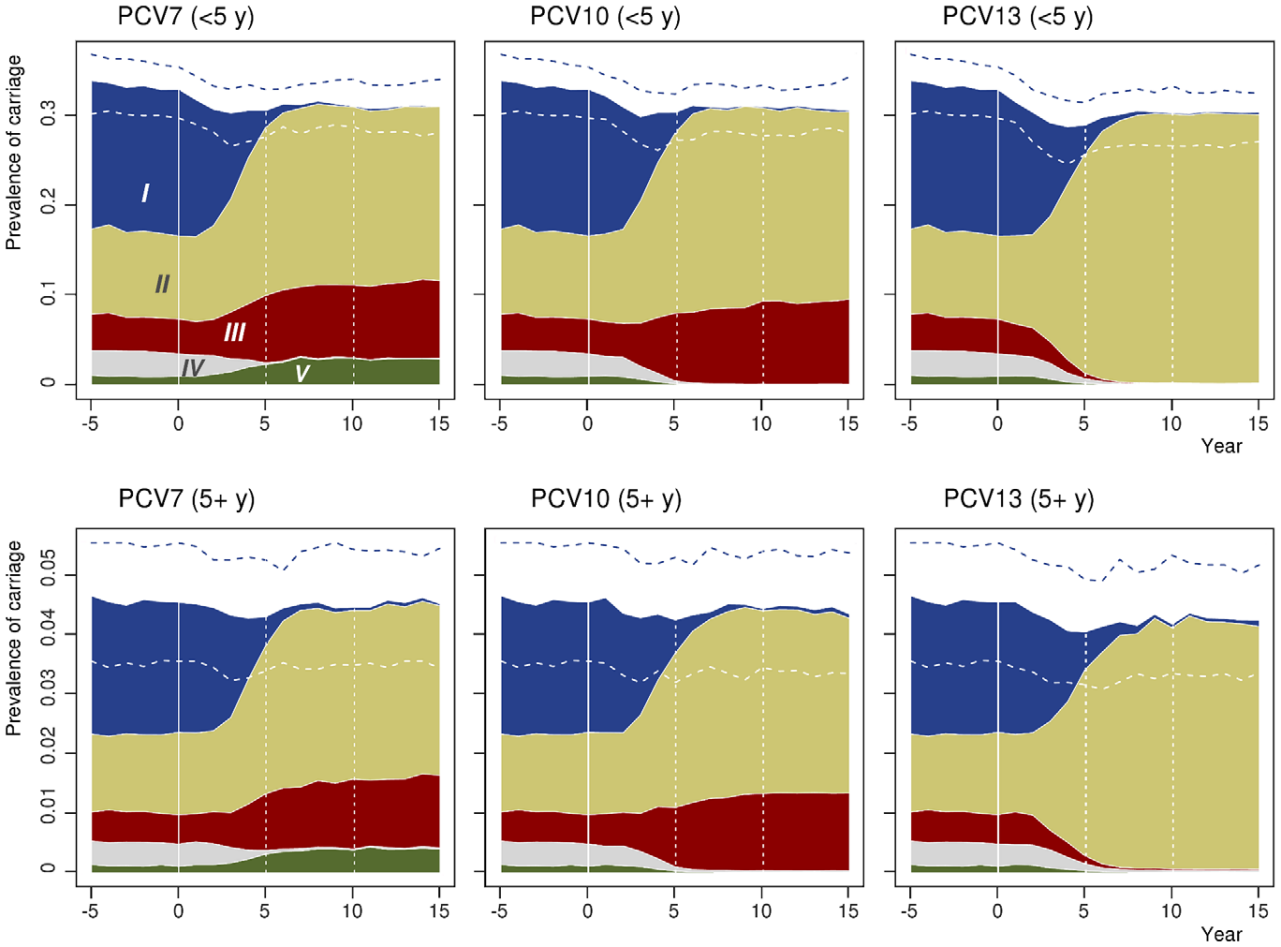
Impact of vaccination on pneumococcal carriage in two age classes in Finland. Each panel presents the projected cumulative prevalence of carriage of the five serotype categories (from V to I) as a function of time, with time 0 corresponding to the onset of the vaccination programme. The point-wise median prevalence based on the 50 most optimal parameter combinations is shown cumulatively by serotype category. For the total prevalence of all five categories, also the 5% and 95% quantiles (dashed curves) are shown. *Upper panels*: individuals <5 years of age. *Lower panels*: individuals 5+ years of age. From left to right: PCV7, PCV10, PCV13. To adjust for a changing demography, the prevalence shown in the figure is standardised according to the age distribution of Finland in 2005. In the simulation, the values of vaccine efficacy against carriage (*φ_c_*) was 0.50 for all vaccine types in the respective vaccine formulation, the coverage of vaccination (*p_c_*) 0.90 and the waning rate of immunity against carriage (*w*) 10% per year.

The simulation model was run for an initial population of 100,000 people consisting of 20 neighbourhoods of equal size and for a period of 60 years prior and 40 years following the implementation of an infant vaccination programme. At the end of each year the age-specific prevalence of carriage of each of the 5 serotype categories was calculated. Figure 6 presents the projected levels of prevalence in two age classes (<5 and 5+ years of age) for the three vaccine formulations (PCV7, PCV10, PCV13). Using the baseline values of vaccine efficacy against vaccine-type acquisition (*φ_c_* = 50%), coverage of vaccination in the childhood programme (*p_c_* = 90%) and the waning rate of vaccine-induced protection (10% per year), vaccine-type carriage is essentially eliminated in the whole population within 5–10 years after the onset of the vaccination programme, suggesting the herd effect taking place in a relatively prompt manner. However, the model projects only a modest (at most 10%) decrease in the overall carriage prevalence as the non-vaccine serotypes effectively replace the eliminated vaccine types in carriage. Moreover, the relative prevalence of the non-vaccine categories remains approximately the same as in the non-vaccinated situation. These results are similar irrespective of the vaccine formulation.

### Thresholds for elimination of vaccine-type carriage

**Figure 7:**
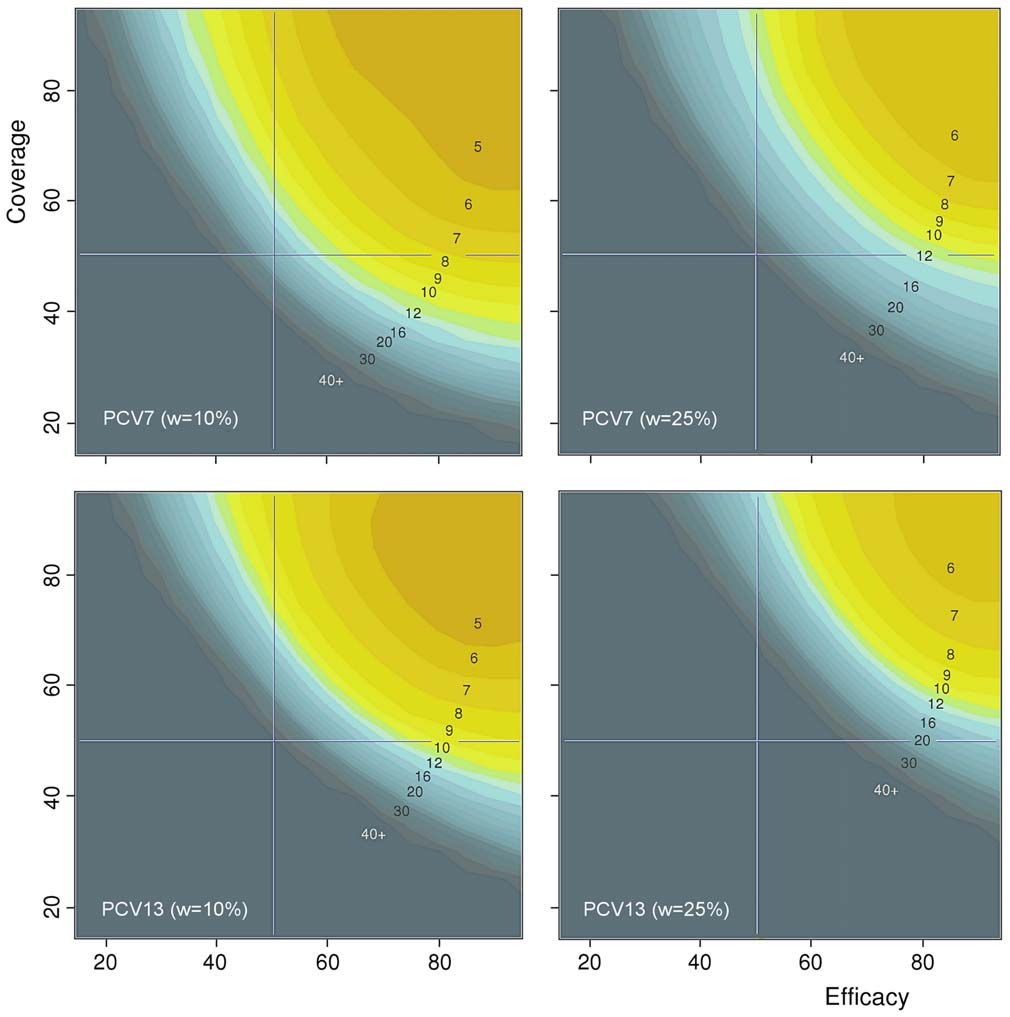
Thresholds for elimination of vaccine-type carriage among children less than five years of age. The figure shows the time in years until near elimination (5% of the level of vaccine-type carriage prevalence in the pre-vaccination era) for PCV7 and PCV13 and for different combinations of the values of vaccine efficacy against carriage (horizontal axis in each plot) and coverage of vaccination (vertical axis). *Left panels*: The waning rate of vaccine-induced immunity against carriage is 10% per year. *Right panels*: the waning rate is 25% per year. *Upper panels*: PCV7. *Lower panels*: PCV13. The numbering within each panel corresponds to lower limits (in years) of the respective colour codes, with 40+ corresponding to 40 or more years until near elimination. The results were obtained using the most optimal parameter combination. For results corresponding to the 50 most optimal parameter combinations, see Figure S3.2 in File 3.

The impact of vaccine efficacy against carriage, coverage of vaccination and the waning rate of vaccine-induced protection against carriage were investigated in a systematic manner by tabulating, for various values of these three quantities, the number of years it takes after the onset of a vaccination programme for the carriage prevalence of vaccine types to reach a level of 5% of the pre-vaccination carriage prevalence in children <5 years of age. Figure 7 presents these results for two PCV formulations (PCV7 and PCV13) and two waning rates (10% and 25% per year). Those sets of values of the two quantities that correspond to effective vaccination programmes are easily detected. The threshold behaviour is similar for PCV7 and PCV13. If the waning rate would be as high as 25% (right panels), elimination becomes more difficult and a high coverage of vaccination is essential. These results about the threshold behaviour were concordant among the 50 most optimal parameter combinations. Interestingly, they also remained essentially the same under the alternative model of stronger between-serotype competition (*θ*_1_ = 0.5) (see Figure S3.2 in File S3).

### Implications of the projected carriage patterns on IPD

Together with the age and serotype category dependent case-to-carrier ratios, the projected levels of carriage predict the incidence of IPD. The lower panels in Figure 4 illustrate the predicted IPD incidence in individuals <5 and 5+ years of age for the three PCV formulations (see also Figure S2.3 in File S2). In individuals <5 years of age, the predicted IPD incidence is approximately 24%, 37% and 71% less than without vaccination for PCV7, PCV10, PCV13, respectively. However, in individuals 5+ years of age, the IPD incidence is much less reduced by vaccination with PCV10 and PCV13 (2% and 23%, respectively) and not reduced with PCV7. This is mainly because vaccination eliminates the category of paediatric serotypes with the lowest case-to-carrier ratios among the adult population.

## Discussion

We constructed an agent-based dynamic transmission model of pneumococcal carriage and disease to explore some of the key determinants of the indirect effects of large-scale vaccination with pneumococcal conjugate vaccines (PCVs). Both of the principal types of indirect effects, herd immunity and serotype replacement, are mediated through the direct protective effect of vaccination on carriage, the source for pneumococcal transmission. Our findings are in agreement with earlier assertions that elimination of pneumococcal disease with serotypes included in the vaccine requires only a moderate direct efficacy against acquisition of carriage (e.g. Lipsitch, 1997). However, even in the event of elimination of vaccine-type (VT) invasive pneumococcal disease (IPD), there will be replacement disease whose incidence greatly depends on the disease causing propensity of the serotypes not included in the vaccine formulation.

This study corroborates to the notion that the transmission potential of pneumococcal carriage is moderate. Based on a susceptible–carrier–susceptible dynamics, we derived an approximate reproduction number *R*_0_ = 2 for an individual pneumococcal serotype. This value is broadly in line with a previous analysis of pneumococcal transmission in a population of mixing groups [30]. Such a low reproduction number implies that carriage of the common serotypes can be effectively controlled by a reasonable direct impact of vaccination on carriage of the respective types, provided the coverage of vaccination is high (Figure 7 and Figure S3.2 in File S3). In a model-based study of the transmission dynamics of pneumococcal carriage and disease, an 86% coverage was enough for elimination of the vaccine types with vaccine efficacy against acquisition of the aggregate vaccine type at 76% and a waning rate of efficacy at 12% per year [18].

Assuming a negligible waning rate, the condition for obtaining herd immunity is *p_c_φ_c_*>1−1/*R*_0_, where *φ_c_* is the vaccine efficacy against carriage and *p_c_* is the coverage of vaccination [35]. This inverse relation between *p_c_* and *φ_c_* is clear also in the current setting (Figure 7) although the required efficacy and coverage are less than implied by the simple formula. This is obviously due to the fact that in a vaccinated population the non-vaccine types gain a fitness benefit which helps them to out-compete vaccine-type colonisation. This assertion is further supported by the finding that the model predictions about vaccine-type elimination did not depend on the assumed level of between-serotype competition (File S3).

With a higher rate of waning (25%), the threshold moves towards higher values of *p_c_* and *φ_c_*. The current knowledge about the duration of vaccine-induced protection against pneumococcal carriage is scarce. In a cross-sectional study among toddlers that had participated in a vaccine trial 3–4 years earlier, vaccine efficacy against VT carriage was approximately 40%, indicating a slow decline in vaccine-induced protection [27].

When elimination of vaccine types is reached, the pattern of serotype replacement in carriage is determined by the competitive interactions among the non-vaccine types (NVT), independently of vaccination-related parameters. Our model of between-serotype competition was constructed so that almost complete replacement of carriage occurs at the post-vaccination equilibrium, i.e., the incidence of NVT carriage in the new equilibrium is close to the overall incidence rate of all-type carriage in the pre-vaccination era. This feature corresponds to empirical observations from populations in which pneumococcal conjugate vaccine has been in large-scale use [14], [15], [36]. Moreover, the relative proportions of the non-vaccine types as causes of carriage retained their pre-vaccination era relative magnitudes. Data from long-term surveillance of some vaccinated populations indicate that this may indeed be at least the first approximation of the distribution of serotypes in the post-vaccination era [36], [37]. Of note, given the ratio of the two competition parameters, the overall level and pattern of replacement in carriage were largely independent of their magnitudes (Figure 6 and File S3).

In addition to the new equilibrium in the incidence of carriage, replacement in disease (IPD) depends on the type- and age-specific case-to-carrier ratios. With increasing valency of the vaccine, the model projects a decreased IPD incidence among the target population of children <5 years of age and a longer time until near-elimination. Because of the different distribution of case-to-carrier ratios among the general population, the expected effect on the overall IPD incidence is milder. In particular, although category I with the three paediatric serotypes is clearly a major cause for IPD among children, it has a low case-to-carrier ratio among the general population compared to the case-to-carrier ratios of the categories of the replacing non-vaccine types.

To our knowledge, our model is the first agent-based dynamic model of pneumococcal carriage and disease which accommodates a large number of individual serotypes. Karlsson [20] considered two aggregate types in an agent-based model to study the role of antibiotic use on pneumococcal transmission. Other approaches have been based on compartmental deterministic models. Effelterre et al. [22] considered the effects of antibiotic use and vaccination in children less than 2 years of age, based on a dynamic model for 18 serotypes or groups. Melegaro et al. [29] investigated the long-term effectiveness of PCV7 vaccination in the UK. Their model was based on pooling the vaccine types and non-vaccine serotypes into two aggregate types and calibrating the efficacy and duration vaccine-induced protection against carriage to produce the post-vaccination pattern of herd immunity and serotype replacement in the US, i.e., in another population after the introduction of PCV7. The calibration of an updated model was based on 3-year post-vaccination surveillance of IPD in the UK [19]. Of note, the model in the present paper was calibrated using pre-vaccination data in a single population. The efficacy and duration of vaccine-induced protection against carriage were free control parameters in post-vaccination projections of carriage and disease (Figure 7).

Clearly, building a dynamic transmission model which would address the more than 90 pneumococcal serotypes individually is both impractical and unnecessary. Some kind of pooling of serotypes is called for. Our approach is based on splitting of both the vaccine and non-vaccine groups of serotypes, corresponding to each PCV formulation, into individual types or subcategories. This feature has important implications. First, pooling serotypes simply into vaccine and non-vaccine types would create two “super-types”, with an overestimated overall rate of acquisition for the vaccine types. This might translate into exaggerated claims of the required vaccination effort for elimination of vaccine-type carriage and disease. Second, the nearly complete replacement of carriage cannot be easily reproduced with a model with only two types. By contrast, our model with 11 competing model types reproduces this phenomenon. Third, any projections on the post-vaccination pattern of IPD based on two types could be biased due to the lack of any detail regarding the rate and pattern of carriage. Furthermore, the additional cost of including more than two types into the model is minimal, with only two parameters required to parameterise the between serotype competition. Our simple empirical model of competition among the serotypes is consistent with the hypothesis that the polysaccharide capsule (i.e., serotype) may affect the fitness of the bacterium in competition with other serotypes during colonisation [17].

The mixing pattern induced by the demographic characteristics implemented in our model emphasizes the importance of contacts among children in families, day care centres and schools and contacts between parents and their children. This is in agreement with strong assortative contact patterns found elsewhere [18], [32], [38].

Potential caveats in our model assumptions include the stability of the serotype distribution by age and the homogeneity of vaccine protection against all vaccine types. The distribution of serotypes was based on data about pneumococcal carriage in children less than 2 years of age (Figure 2B; [25]). It is possible that the dominant serotypes are different in older children or adults [36] although the scarcity of data declined a conclusive analysis of this in the current setting. The threshold for VT elimination is likely to be robust to slight misspecifications of the serotype distribution in adults because of the importance of the younger age classes in sustaining transmission. By contrast, the projected IPD incidence in adults could be more prone to bias due to biased rates of acquisition and case-to-carrier ratios. For example, if the actual proportion of non-vaccine serotypes in adults is larger than in the model, the corresponding case-to-carrier ratios in our analysis would be over-estimates. This would lead to exaggerated predictions on replacement disease in adults so that the 22% increase in IPD under use of PCV7 (Figure 4) would be pessimistic. This problem is likely to be largest with PCV7, which covers serotypes commonly carried by children. An analogous argument applies to biases induced by the assumed similarity of serotype-specific clearance rates. For example, if some non-vaccine serotypes are carried for clearly shorter times than in the model, the estimated acquisition rates would be under-estimates and, subsequently, the case-to-carrier ratios over-estimates. This could lead to over-estimates of replacement disease by such serotypes.

The vaccine efficacy against acquisition of carriage was assumed to have the same value for all vaccine serotypes. Although there maybe differences in efficacy between individual serotypes, the overall efficacy against serotypes in various PCV formulations appears to be somewhere about 50% [39]. The current findings can therefore be expected to hold for most vaccine serotypes. Vaccine-induced cross-protection was not considered in the model although some serotypes may be affected by cross-reactive immunity, serotype 6A being the most frequently carried and an important source of replacement. However, the data in this study did not differentiate between serotypes 6A (potential cross-immunity) and 6C (no cross-immunity) which were formerly both classified as 6A [40]. The inclusion of 6A as an NVT in the analyses for PCV7 and PCV10 is likely to lead to too low IPD predictions, based on the relative importance of 6A as cause of carriage and its low case-to-carrier ratio. The possibility for capsular switching was also not featured in our model. However, its impact on pneumococcal population has been suggested to be minor in comparison to serotype expansion caused by reduced competition by the vaccine types [41].

The vaccine-induced protection was not assumed to take effect until the booster dose at 12 months of age. This model feature excludes the possibility to address questions about the impact of different schedules and dosage of vaccination. However, model-based predictions about the indirect effects and the net effectiveness of vaccination depend on the assumed vaccine efficacy against acquisition and the waning rate of protection. Assuming protection only after 12 months represents a conservative approximation, should there be considerable protection starting during the earlier phase of the schedule.

One of the key questions about the long-term effectiveness of pneumococcal vaccination concerns the extent and pattern of serotype replacement in different disease manifestations. In this study, of the possible manifestations of pneumococcal disease, we only considered invasive pneumococcal disease. This was because of the relatively sensitive and specific data on the incidence of IPD in Finland. Acute otitis media (AOM) and mild respiratory infections are probably not of interest because of rapid replacement of carriage in the long-term. The question of pneumococcal pneumonia remains to be tackled, in particular in developing country settings where it is thought to constitute the main burden of pneumococcal disease. The current micro-simulation approach offers a suitable platform for such extensions.

## Supporting Information

File S1 **The demographic model. Transmission by age and mixing group. Tables S1.1 and S1.2.**
(PDF)

File S2 **Supplementary information on model calibration, parametrisation and the likelihood function. Texts S2.1 and S2.2. Figures S2.1–S2.3.**
(PDF)

File S3 **Model projections under alternative parameter values. Figures S3.1 and S3.2.**
(PDF)
