# Supplementary material for: Pneumococcal Transmission and Disease In Silico: A Microsimulation Model of the Indirect Effects of Vaccination
Source: PLoS One. 2013 Feb 6;8(2):e56079. doi: 10.1371/journal.pone.0056079 (PMC3566073; doi:10.1371/journal.pone.0056079)
Supplement: File S1 — The demographic model. Transmission by age and mixing group. Tables S1.1 and S1.2. (PDF) [file pone.0056079.s001.pdf]

File S1

The demographic model. Transmission by age and mixing group. Tables S1.1 and S1.2.

Table S1.1

Mixing groups and possible life history events in the demographic model.

A. List of the mixing groups in the population model, their sizes (number of persons) and ranges for the age of individuals (in years) belonging to each group.

B. Summary of possible life history events during the lifespan of an individual. Some events only apply to a fraction of the individuals.

The population in the demographic simulation model mimics that of Finland and is based on a platform previously utilised in [Ref1]. Detailed statistics from Finland [Ref2] were used for the construction and calibration of the population model.

A

Type	Size	Age (y)	Explanation
Family (*)	1-10	0-100	4 types of familes:<br>(a) Single person aged 20-100 (50% of families)<br>(b) Couple with children aged 0-19 (**)<br>(c) Single parent with children<br>(d) 2 adults only (20%)
Day Care (type 1)	3-6 (65%)<br>6-13 (35%)	0-2	65% of day care attendees in family day care<br>35% in day care centres (larger group size)
Day Care (type 2)	3-6 (65%)<br>13-23 (35%)	3-6	Day care for older children<br>(average group size larger)
School	10-15 (***)	7-16	Class size 20-30.<br>Each school-aged child belongs to a school.
Neighbour-hood (*)	appr. 5000	0-100	Population of 100 000 divided<br>into 20 neighbourhoods

(*) Each individual belongs to one family and one neighbourhood.
(**) Average number of children: 1.8.
(***) Number of classmates relevant to transmission is used in the model.

B

Event	Birth	Enter day care	Enter school	Move from home
Age (y)	0	8 -18 months (if applicable)	7	20
Expla-nation	(§)	Randomly selected 44% attend day care.<br>Entrance at a random age (8-18months).	All children attend school.	Child leaves parental household and forms a single person family.

Event	Marriage	Divorce	Death
Age (y)	20-100 (if applicable)	20-100 (if applicable)	0-100
Expla-nation	Single (or single-parent) individuals marry persons within same age group at an age-dependent rate.	Family is divided into two: one single person family and one single parent with children.	(§§)

(§) Of the newborns 51.5% are male. Single women do not give birth in the model.
(§§) Life expectancy is 77 and 84 years for men and women respectively.

Table S1.2

Proportion of transmission events by age and mixing group.

A. Transmission events between five age classes as a proportion (%) of the total number of transmission events per time unit.

B. Transmission events within each of the five mixing groups as a proportion (%) of the total.

The value listed in each cell corresponds to the most optimal parameter combination and the range in parentheses is the plausible range from simulations using the 50 most optimal parameter combinations. Each value is based on the stationary stratum specific age distribution of susceptibles recorded at year 50 of the simulation and on a contact matrix calculated for a stratified population with 55 strata according to age, presence of siblings, day care attendance status and parenthood status (see the last paragraph of section "Structure of transmission").

A

Age of recipient	Age of transmission source: 0-6	7-19	20-44	45-64	65+
0-6	18.2 %<br>16.7-30.8	2.1<br>1.2-2.4	12.8<br>6.1-17.6	0.2<br>0.1-0.3	0.1<br>0.0-0.1
7-19	4.6<br>3.7-6.1	11.7<br>4.1-19.0	3.9<br>2.4-4.8	1.6<br>1.0-1.9	0.1<br>0.0-0.2
20-44	17.9<br>15.6-21.7	6.0<br>3.3-6.7	6.9<br>4.5-8.0	0.6<br>0.4-0.8	0.1<br>0.0-0.3
45-64	2.1<br>1.6-3.4	3.8<br>2.1-4.3	1.3<br>1.0-1.7	2.3<br>1.5-2.8	0.3<br>0.2-0.4
65+	1.1<br>0.6-2.1	0.5<br>0.2-0.9	0.7<br>0.4-1.1	0.4<br>0.2-0.6	1.0<br>0.6-1.3

B

Site of transmission: Family	Day care 1	Day care 2	School	Population
69.9 %	3.1	6.7	7.4	12.8 *
44.5-80.4	1.1-9.3	4.3-12.9	1.5-14.9	7.3-22.6 *

* General population and neighbourhood combined.

References for Table S1.1

[Ref1] Auranen K, Eichner M, Leino T, Takala AK, Makela PH, et al. (2004) Modelling transmission, immunity and disease of haemophilus influenzae type b in a structured population. Epidemiol Infect 132(5): 947-957.

[Ref2] Statistical yearbook of Finland 1995 (in Finnish), Statistics Finland.
