# Supplementary material for: Pneumococcal Transmission and Disease In Silico: A Microsimulation Model of the Indirect Effects of Vaccination
Source: PLoS One. 2013 Feb 6;8(2):e56079. doi: 10.1371/journal.pone.0056079 (PMC3566073; doi:10.1371/journal.pone.0056079)

File S2

Supplementary information on model calibration, parametrisation and the likelihood function. Texts S2.1 and S2.2. Figures S2.1 - S2.3.

Text S2.1 The Likelihood function

Text S2.2 Calibration of the simulation model

Rates of acquisition, Competition parameters,
Calibration design, Model fit and stochastic variation

Figure S2.1 Profile likelihood function and the 50 most optimal parameter value combinations.

Figure S2.2 Log-likelihood values at the 400 best parameter combinations

Figure S2.3 Projected IPD incidences from repeated simulation runs

Text S2.1

The likelihood function.

The simulation model was calibrated to empirical data on the age-specific prevalence counts y_{1i}, i = 1,…, N_1, in N_1 = 8 age groups (Figure 2A) and the serotype-specific prevalence counts y_{2i}, i=1,…, N_2, in N_2 = 5 serotype categories among children less than 2 years of age (Figure 2B) prior to PCV vaccination. The corresponding sample sizes for the age-specific prevalence counts are denoted by n_{1i}, i=1,…, N_1. Denote the model parameters (Table 1) by α. The model outputs for a given vector α are the vectors of age-specific carriage prevalence in the 8 age groups and the serotype distribution in the 5 categories, denoted by $p_1(\alpha)$ and $p_2(\alpha)$, respectively. The model output was determined as the average of cross-sectional samples of pneumococcal carriage taken from 10 years at the end of a 60-year simulation.

The age-specific prevalence counts are mutually independent binomial samples so that their likelihood contribution is

$$L_1(y_{11}, y_{12}, \dots, y_{1N_1} \mid p_1(\alpha)) = \prod_{i=1}^{N_1} \binom{n_{1i}}{y_{1i}} p_{1i}^{y_{1i}} (1-p_{1i})^{n_{1i}-y_{1i}} \quad .$$

The serotype-specific prevalence counts are a sample from a multinomial distribution and the likelihood contribution is

$$L_2(y_{21}, y_{22}, \dots, y_{2N_2} \mid p_2(\alpha)) = \begin{pmatrix} y_{21} + y_{22} + \dots + y_{2N_2} \\ y_{21}\ y_{22}\ \cdots\ y_{2N_2} \end{pmatrix} \prod_{i=1}^{N_2} p_{2i}^{y_{2i}} \ .$$

The joint likelihood function for the parameter vector α, based on both types of data, is thus given by the product

$$L_1(y_{11}, y_{12}, \dots, y_{1N_1} \mid p_1(\alpha))\ L_2(y_{21}, y_{22}, \dots, y_{2N_2} \mid p_2(\alpha)) \,. \qquad (1)$$

Text S2.2

Calibration of the simulation model.

The simulation model involves 11 parameters related to acquisition (Table 1): 3 age-specific rates of acquisition, 6 mixing group-specific relative rates of acquisition and 2 competition parameters. The values of these parameters were assigned in the context of the model. For definiteness, one of the rates of acquisition (β_1) was assigned an arbitrary value. Moreover, assuming fixed values for more than just one of the parameters was necessary as calibrating the model with respect to a 10 dimensional parameter space would have been impractical.

■ Rates of acquisition ($\beta_1, \beta_2, \beta_3$)

The two groups of rates of acquisition (the age-specific and the mixing group-specific rates) are strongly interlaced and estimation of both of these groups of parameters causes identifiability problems which complicate the calibration of the model. For this reason, we set the 3 age-specific rates of acquisition to fixed values. To determine these values and to find reasonable ranges for the rest of the parameters, we conducted a series of exploratory calibration runs, where the model output corresponding to various parameter value combinations was compared to the observed data in Figure 2. In doing this, 4 of the mixing group-specific relative rates (family, school and the 2 day care rates) were all initially assumed to be approximately 1. Based on the results from these runs, we assigned the 3 age-specific rates of acquisition ($\beta_1, \beta_2, \beta_3$) the fixed values given in Table 1. These values are roughly proportional to 5, 7 and 2 for under 1 years olds, 1-3 years olds, and 4 year olds and older, respectively, and they reflect reasonable differences in age-specific per contact susceptibilities among individuals. The relative rate for the population was arbitrarily chosen to be half of that for the neighbourhood, meaning that two thirds of the infectious contacts the individual makes in the general population (neighbourhood and the population combined) are with individuals in the same neighbourhood.

■ Competition parameters (θ_1, θ_2)

As the competition parameters θ_1 and θ_2 were expected to be heavily dependent on each other, the calibration was performed using the transformed parameters θ_1 and θ_2/θ_1. The parameter θ_1 determines the level of double carriage in the model whereas the ratio θ_2/θ_1 is closely related to the extent of competition and largely determines the shape of the serotype distribution in carriage. After the exploratory calibration runs, it was clear that the optimal value of θ_2/θ_1 is well calibrated with the optimal value 0.93 and, for those parameter value combinations where the model fit is fairly good, is also independent of other parameter values. On the other hand, the range of

values for θ_1 was found to be only roughly identifiable, supporting both low and high levels of double carriage. Obviously, this is due to the fact that the calibration data have no information on double carriage. Consequently, we set the value of θ_2/θ_1 to 0.93 and allow two values for θ_1. We chose these to be 0.5 (moderate double carriage; 9-22% of carriers) and 0.8 (high double carriage; 15-30% of carriers) and performed further calibration of all of the other remaining 5 parameters (the mixing group-specific relative rates) separately under the moderate and high double carriage assumptions.

Calibration design

In the final calibration, each simulation run consisted of a population of 100 000 individuals (5000 in each of the 20 neighbourhoods). The demographic details of the simulated population were initialised with the Finnish population statistics and the infection process with the age-specific and serotype-specific data on carriage prevalence, not stratified by other aspects of the population structure. During the calibration process the length of each simulation run was 60 years. Corresponding to the observed data (Figure 2), 13 values (8 age-specific prevalences and 5 serotype proportions) were recorded and averaged over the years 51-60 of the simulation. The calibration was conducted with respect to the 5 relative rate parameters parameters (d_1, d_2,… d_5) and included 3 consecutive designs for the parameter value combinations. The design for each new set of parameter value combinations was determined based on the calibration fit from the preceding designs. The first and the third design included 3 levels for each of the 5 parameters in a Cartesian product manner, each consisting of 243 (=3^5) design points. The second design consisted of 3 levels for 3 of the parameters and 2 levels for 2, thus constituting 108 (=$3^3 \times 2^2$) design points. The total number of design points (parameter value combinations) was thus 594. For each design point, 13-18 repetitions were realised. This constituted a total of approximately 9000 computer simulation runs separately for both the low and high values of θ_1.

For the 5 relative rate parameters, the most optimal parameter combinations were defined as those corresponding to the 50 largest median likelihood values among the repeated simulations. The pair-wise scatterplots of the 50 most optimal parameter combinations are shown in Figure S2.1.

Model fit and stochastic variation

For each simulation run, the logarithm of the likelihood value (1) was evaluated and for each design point the median log-likelihood value from the corresponding simulation runs was recorded as a measure of model fit. In addition, to assess the amount of stochastic variation in the simulation model, the shortest intervals covering 50% and

75% of the log-likelihood values were recorded for each design point. Figure S2.2 shows the median log-likelihood values and the 50% and 75% coverage intervals corresponding to the 400 best fitting parameter value combinations for θ_1=0.8. The quantities related to the first part of the likelihood function (1), corresponding to model fit to the age-specific prevalences, are plotted separately. Clearly, among the most optimal parameter combinations, the stochastic variation in the simulations is mostly related to the serotype distribution whereas the age-specific prevalences are very similar among repeated simulation runs corresponding to the same design point.

The stochastic variation among the projected levels of carriage and disease was assessed by studying results from the approximately 15 repeated simulation runs for each of the 50 most optimal parameter combinations. Figure S2.3 shows the projected steady state IPD incidences following PCV10 and PCV13, separately for 2 age categories (cf. Figure 4). Figure S2.3 suggests that most of the 50 best parameter combinations produce similar average predictions. Also, the level of stochastic variation is similar. Based on this and other similar comparisons (not shown) we conclude that the stochastic variation shown in Figure S2.2 does not translate into notable stochastic variation in most of our key predictions.

Figure S2.1

Profile likelihood function and the 50 most optimal parameter value combinations.

The background colouring indicates levels of approximate profile likelihood functions with dark colouring (yellow and red) corresponding to high values and light colouring (white and grey) to low values. The pair-wise likelihood functions are shown with respect to each of the five calibration parameters: d_1 (family), d_4 (school), d_2 (day care 1), d_3 (day care 2) and d_5 (neighbourhood). The likelihood function approximation is based on a local polynomial regression model*. In addition, pair-wise scatterplots of the 50 most optimal parameter combinations are superimposed. A small amount of random noise is added to make overlapping points visible. The most optimal parameter combination (Table 1) is indicated by a red cross, those ranked among the best 15 parameter combinations by large circles and the rest by small circles. The range on each axis corresponds to the range of the 594 parameter value combinations used in the final calibration (see section Calibration design in File S2).

* The local polynomial regression fit was calculated using data consisting of the 594 median log-likelihood values and the corresponding design points. The predictive reliability was assessed through repeated 10-fold cross-validation and an optimal smoothing parameter value was chosen. For the optimal value, 100 sets of 594 predicted values were obtained. Among the 50 most optimal parameter combinations, the difference between the actual and the predicted log-likelihood value was less than 20 in absolute value with frequency 94%. (The actual log-likelihood values are shown in Figure S2.2.) Among the 100 best points this frequency was 93% and among the 400 best points it was 88%. Among the design points outside of the 400 best, a predicted likelihood value was among the 300 best with frequency 0.05%. In view of these results the regression model was deemed a satisfactory approximation of the log-likelihood function for the purpose of Figure S2.1.

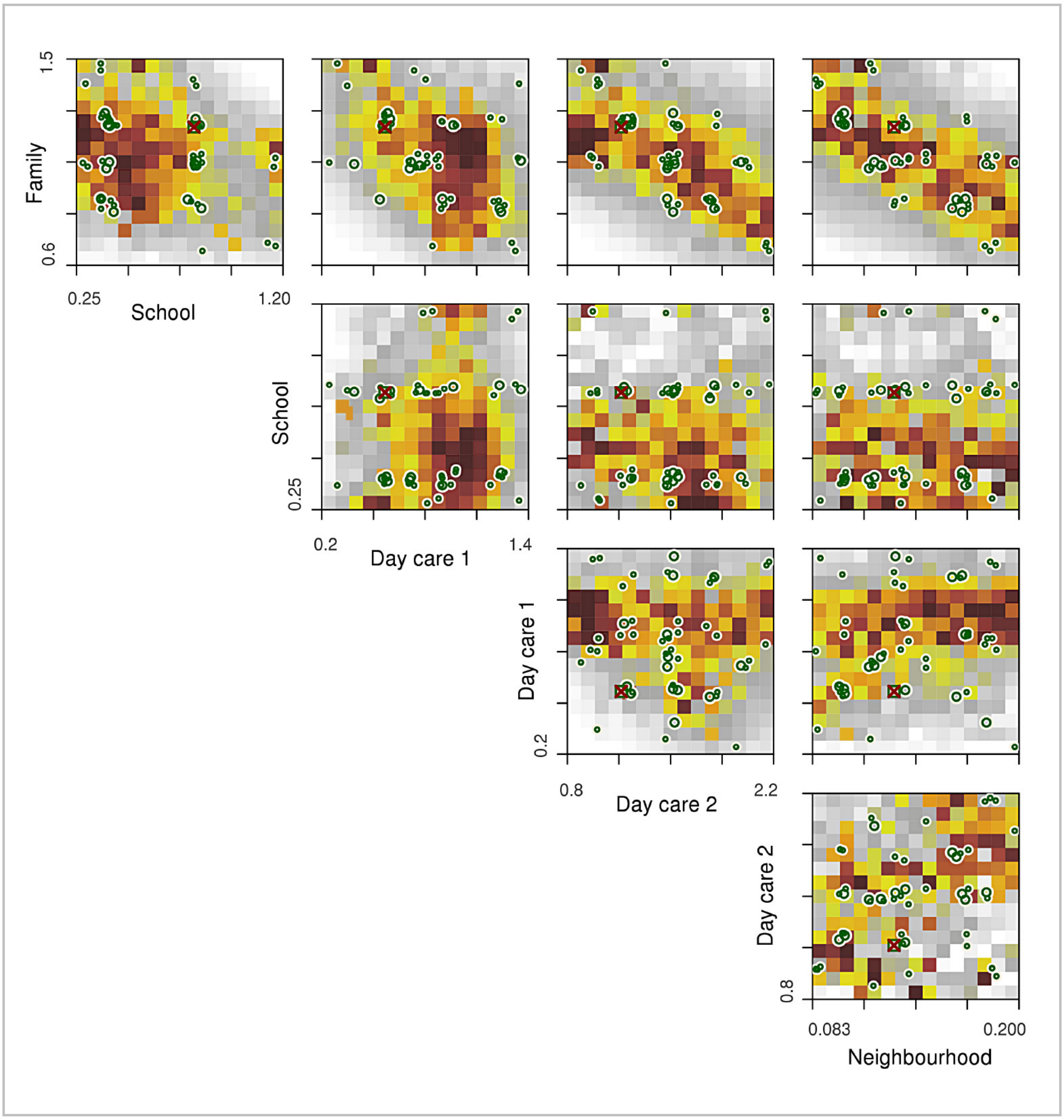

Figure S2.1.

Profile likelihood function and the 50 most optimal parameter value combinations.

Figure S2.2

Log-likelihood values at the 400 best parameter combinations.

Upper part of the figure: The curve (black colour) corresponds to the median log-likelihood values (multiplied by -1) at the 400 best parameter combinations. The vertical lines correspond to the point-wise 50% (orange colour) and 75% (grey) coverage intervals based on approximately 15 repeated simulations at each parameter combination. Lower part of the figure: Median log-likelihood values (red colour) and 75% coverage intervals (blue) corresponding to the first part of the log-likelihood function (1), which is related to the age-specific prevalences.

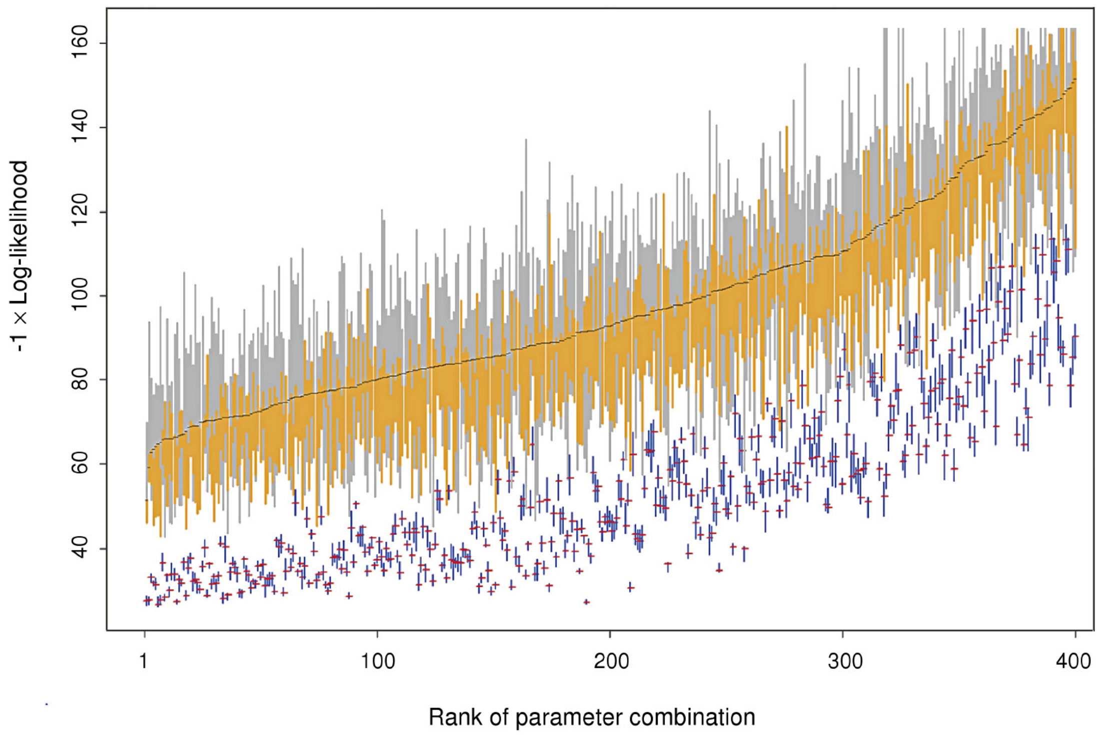

Figure S2.3

Projected IPD incidences from repeated simulation runs.

Projected steady state IPD incidences (per year and per 100 000 individuals) calculated from 13-18 repeated simulation runs corresponding to each of the 50 most optimal parameter combinations. IPD incidence is shown on the vertical axis and the rank of the parameter value combination on the horizontal axis. The black marks indicate the IPD incidences corresponding to the simulation runs related to the 50 largest median likelihood values (i.e. the range of plausible values reported in Figure 4). Vertical lines correspond to the shortest intervals covering 90% (grey colour) and 50% (orange) of the projected IPD incidences calculated from repeated simulation runs at each parameter combination. The 4 panels show results related to vaccine formulations PCV10 (top panels) and PCV13 (bottom panels) for children under 5 years of age (left panels) and for the rest of the population (right panels).

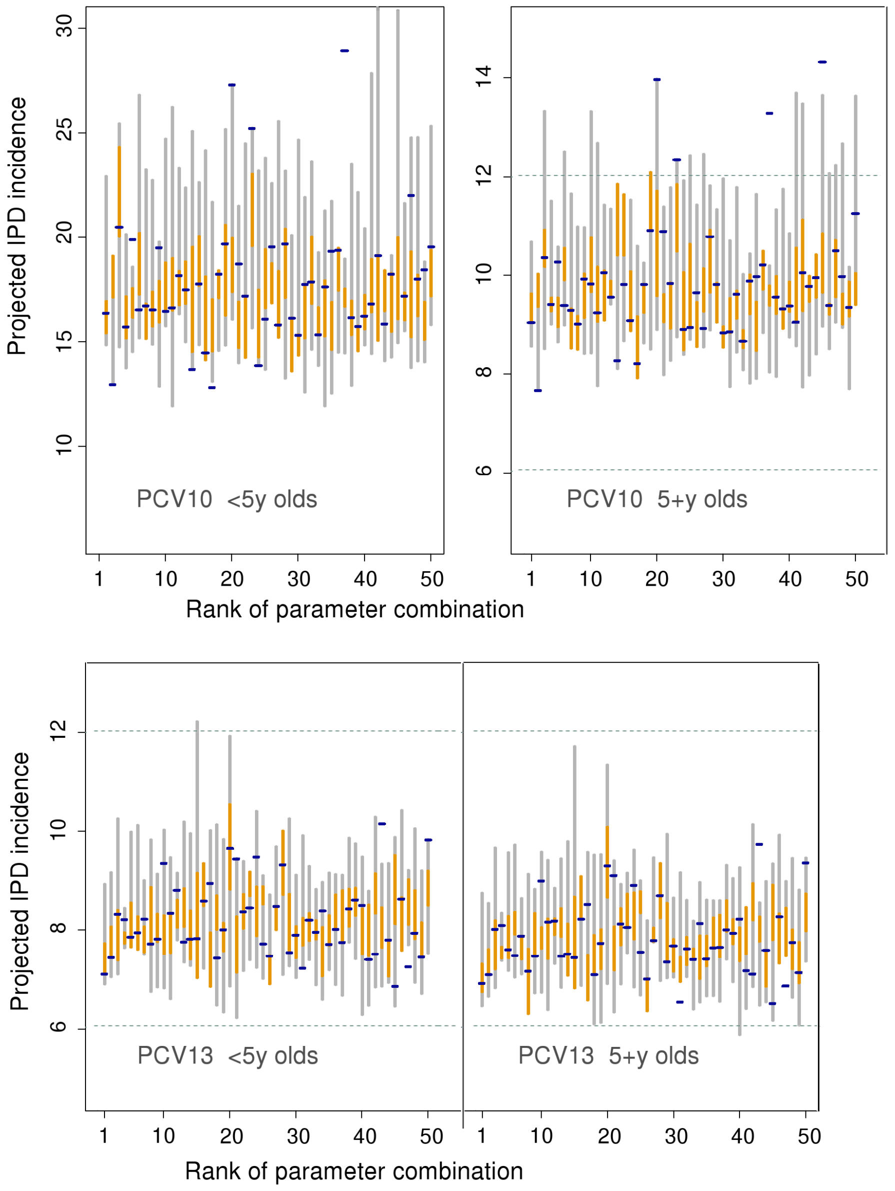

Supplement: File S2 — Supplementary information on model calibration, parametrisation and the likelihood function. Texts S2.1 and S2.2. Figures S2.1–S2.3. (PDF) [file pone.0056079.s002.pdf]
