# Supplementary material for: Pneumococcal Transmission and Disease In Silico: A Microsimulation Model of the Indirect Effects of Vaccination
Source: PLoS One. 2013 Feb 6;8(2):e56079. doi: 10.1371/journal.pone.0056079 (PMC3566073; doi:10.1371/journal.pone.0056079)

File S3

Model projections under alternative parameter values. Figures S3.1 and S3.2.

Figure S3.1

Impact of vaccination on pneumococcal carriage under alternative parameter values.

An alternative calibration fit was obtained using a smaller value (0.5) of the competition parameter θ_1 (for details on calibration see File S2). As in Figure 6, each panel in this figure presents the projected cumulative prevalence of pneumococcal carriage among children <5 years of age in Finland corresponding to the five serotype categories (from V to I) as a function of time, with time 0 corresponding to the onset of the vaccination programme. From left to right: PCV7, PCV10, PCV13. Upper panels: θ_1=0.8. Lower panels: θ_1=0.5. The predicted median prevalence with the 5% and 95% (dashed curves) and the 25% and 75% (solid curves) point-wise quantiles of the cumulative prevalence is shown. In addition, the corresponding quantiles are shown for the sum of the prevalences for serotype categories III, IV and V (lower set of curves

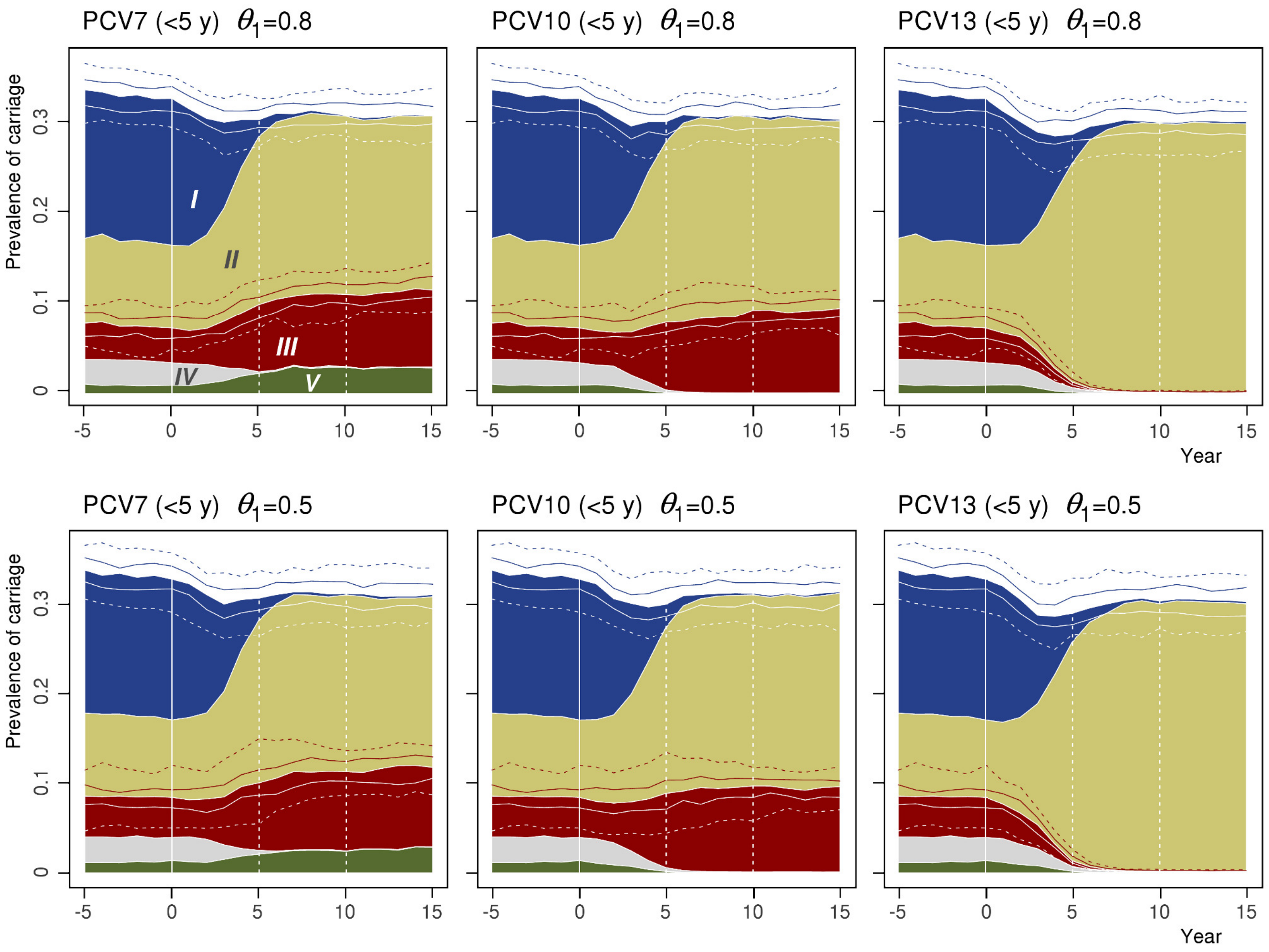

in each panel). The predictive intervals in this figure are based on simulation runs corresponding to the set of 50 most optimal parameter combinations obtained separately for θ_1=0.5 and θ_1=0.8. In the simulation, the values of vaccine efficacy against carriage was 0.50 for all vaccine types in the respective vaccine formulation, the coverage of vaccination (p_c) 0.90 and the waning rate of immunity against carriage (w) 10% per year.

Figure S3.2

Effect of parameter uncertainty on thresholds for elimination of VT carriage for PCV13.

The time in years after the onset of a vaccination programme until near elimination of vaccine-type carriage (see Figure 7) was obtained for the set of 50 most optimal parameter value combinations separately for the competition parameter values θ_1=0.8 (left panel) and θ_1=0.5 (right panel). Near elimination corresponds to 5% of the level of vaccine-type carriage in the pre-vaccination era. Results are shown for children less than five years of age and corresponding to PCV13. The vertical axes indicate the time in years until near elimination. The scale on the vertical axis on each panel is logarithmic while the numbering refers to the original scale. The horizontal axes indicate coverage of vaccination. The time until near elimination was calculated at 5 levels of coverage of vaccination (p_c): 50, 60, 70, 80 and 90%. The results were obtained assuming that vaccine efficacy against carriage (φ_c) is 50% and waning rate of immunity against carriage (w) 10% per year. Point-wise quantiles calculated from the 50 simulation runs are shown and the results corresponding to the 50 optimal parameter combinations are indicated by blue circles.

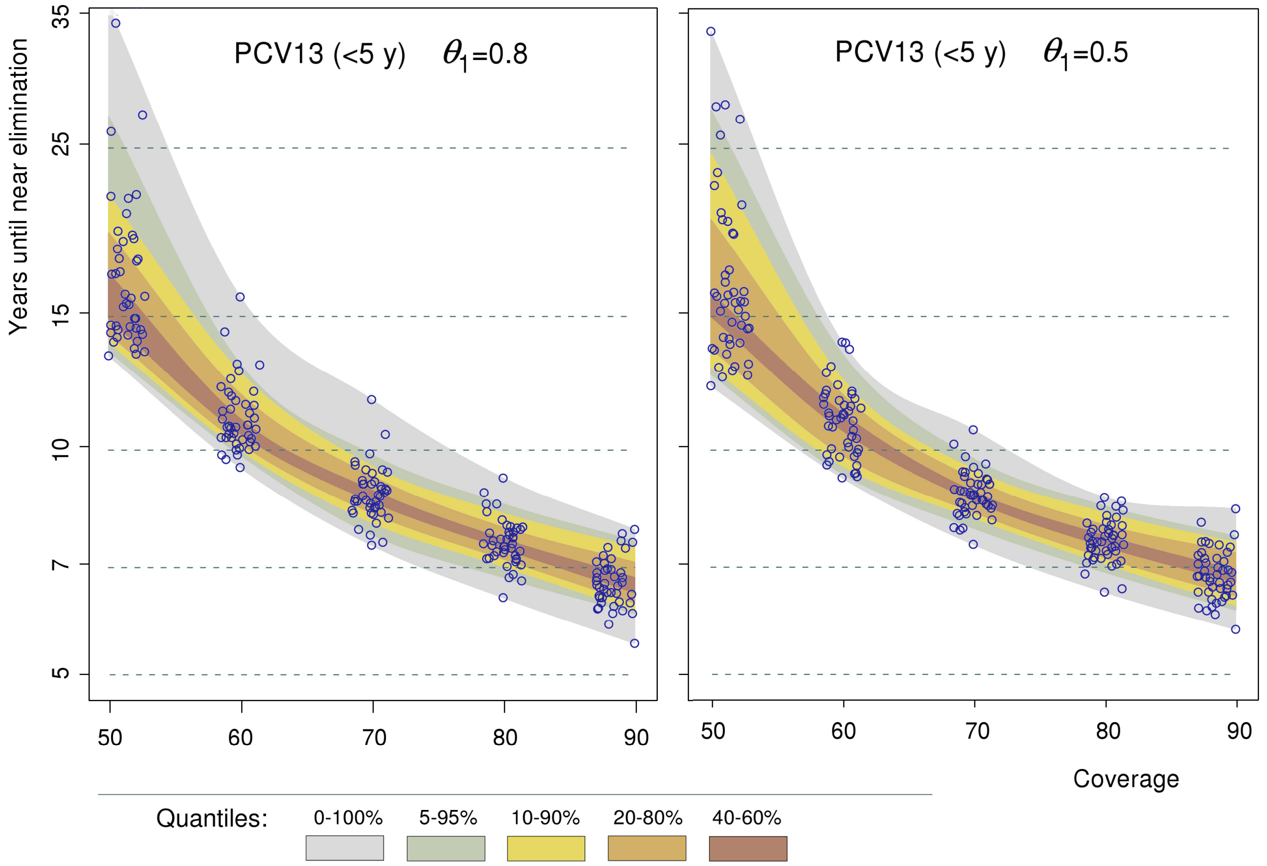

Supplement: File S3 — Model projections under alternative parameter values. Figures S3.1 and S3.2. (PDF) [file pone.0056079.s003.pdf]
